# Acute Rheumatism and Pneumonia

**Published:** 1908-06-27

**Authors:** 


### Medicine.

### ACUTE RHEUMATISM AND PNEUMONIA.

It is well-known that, although lobar pneumonia as a disease has certain well-defined characteristics which are more or less common to all cases and to all epidemics, nevertheless there are peculiarities in the illness which vary in different years. Sometimes all the cases run a mild course, patient after patient reaching the crisis on or before the fifth day. Another year, the reverse is true, the patients being much more severely ill, and the crisis not coming before the tenth day in a great many of them.

### Joint Pains with Lobar Pneumonia.

During the latter part of 1907 there was a peculiarity in the disease which had not been observed to any great extent in former years—namely, the coincidence of more or less severe joint pains with the pneumonia. In one large hospital there were four patients in the wards at the same time, in each of whom the initial symptom was acute pain flitting from joint to joint in such a manner that acute rheumatism was the diagnosis first made; moreover, in one of these cases, a girl aged 16, there were loud bruits indicative of old-standing aortic and mitral disease due to two previous attacks of acute rheumatism. In all four of these apparently rheumatic cases the patient had been but a day or two in the wards when the physical signs of pneumonia developed in the lungs.

Question at once arose as to the nature of this pneumonia. Had the patient started with an ordinary acute rheumatism, and at the same time developed a pneumococcal infection of the lung? Was the whole of the trouble pneumococcal, joint pains and lungs consolidation alike, without any real acute rheumatism at all? Was it lobar pneumonia due to some organism other than the pneumococcus? If so, was the pneumo-bacillus at work both in the joints and in the lungs? Or was the causal organism of both lesions the influenza bacillus? Or was the entire trouble rheumatic, that is to say, were both the joint pains and the lung consolidation due to the supposed organism of rheumatic affections, the diplococcus rheumaticus of Poynton and Paine?

We cannot do more than speculate upon the above possibilities at present; for no detailed bacteriological examinations were possible. It is possible that others may have opportunities for making them in the future.

It is well known, of course, that many different micro-organisms may give rise to that which is clinically lobar pneumonia. Besides the pneumococcus of Frænkel, the pneumo-bacillus of Friedländer, and the influenza bacillus, mentioned above, it has been conclusively shown at St. Mary's Hospital that the lobar pneumonia which sometimes arises during typhoid fever may be due entirely to the bacillus typhosus, which abounds in microscopical sections of the solidified lung, and is cultivable in pure culture. It is, therefore, by no means impossible that the diplococcus rheumaticus may be added to the list, and that a true rheumatic lobar pneumonia may be recognised.

### The Case for a Rheumatic Origin.

The reasons for suspecting that the cases in question were not pneumococcal, but rheumatic, are briefly as follows:

1. In each case the joint pains were the first symptom, and they were clinically indistinguishable from the joint pains of acute rheumatism.

2. The joint pains were relieved by the administration of salicylates. The temperature did not come down with the giving of these drugs, but this does not prove that there was more than a rheumatic process at work; for it is only in straightforward acute rheumatism that the pyrexia ceases when salicylates are given. The pyrexia persists in spite of salicylates when certain accompaniments of acute rheumatism are present; for instance, rheumatic tonsillitis, or rheumatic pleurisy with effusion: it would not be unlikely that the same would apply to rheumatic pneumonia.

3. Several of the patients had been subject to former acute rheumatism, and one at least had well marked valvular heart disease.

4. The patients were much less ill than is the rule with pneumococcal pneumonia; even when the consolidation was bilateral there was comparatively little distress.

5. The patients' temperatures were seldom high, and the charts were not at all typical of ordinary pneumonia. The highest temperature recorded was 103.2° F., and in some of the cases it did not exceed 101° F. A marked feature of the charts was their irregularity.

6. The respiration rate tended to be very much less increased than is the rule in pneumococcal pneumonia. The rate was much nearer 30 per minute than 40, as a rule.

7. The pulse rates on the other hand tended to be much more rapid than in ordinary lobar pneumonia. It was frequently 120, and in the case with aortic and mitral disease it was 132, and yet recovery followed without any untoward severity in the illness. The ratio of respiration rate to pulse rate was thus not nearly so abnormal as it is in most cases of ordinary lobar pneumonia.

8. Not one of the cases had any rusty sputum; indeed, expectoration was in each instance very slight, and when any sputum was brought up it was frothy and light, like that of bronchitis rather than that of pneumococcal pneumonia.

It is, as we have said, only a suggestion that these are cases of acute rheumatic pneumonia, and it is for the future to decide whether rheumatic pneumonia really occurs, or whether the diagnosis should not rather be one or other of the various alternatives enumerated. In any case, however, the clinical condition is sufficiently striking to merit particular attention, even if the lesion turns out to be really pneumococcal pneumonia associated with acute, but fleeting, joint pains that are relieved by salicylates.

